# Letter from John A. Kennicott, M. D., the Grove, Ill.

**Published:** 1861-10

**Authors:** John A. Kennicott

**Affiliations:** The Grove


### Correspondence.

### MILK SICKNESS.

TO THE EDITORS OF THE CHICAGO MEDICAL EXAMINER.

GENTLEMEN:—The letter on "milk sickness," which I send you (for extract or comment in the MEDICAL EXAMINER, if you see fit), is so nearly like my own deductions in its facts, that I deem Mr. Fisher worthy of credit in his hypothesis,—though he submits his remarks to the State Agricultural Society, through my son, Robert, and not to the Faculty, as you will see. While a member of the State Agricultural Board, I made inquiries of intelligent stock-growers, practically acquainted with "milk sickness," and nearly all the facts I then gathered, are repeated in this letter.

Robert Kennicott, while in the southern part of the State, collecting specimens of natural history, ascertained the existence of *cobalt*, in some badly infected districts, but finding no traces of this mineral elsewhere, in localities where the disease existed, he did not publish his discovery.

I have been inclined to believe the "milk sickness" dependent on local miasmatic influence and unwholesome water, rather than any known and tangible mineral or vegetable poison. I have reliable information of the existence of "milk sickness," to an alarming extent, at one time, only forty or fifty miles southwest of Chicago; but never north of there,—while the *cicuta* was once very abundant in the prairie sloughs of this county, and is still plentiful hereabouts, though not where cattle drink. Any remarks from your pen would, I am sure, be welcome to reading stock-growers, in the few localities of this State where cases of this inexplicable affection still occur.

Your friend,

JOHN A. KENNICOTT, M. D.

THE GROVE, August, 1861.

